# Human Papillomavirus (HPV) Prevalence and Type Distribution in Urban Areas of Malaysia

**DOI:** 10.31557/APJCP.2021.22.9.2969

**Published:** 2021-09

**Authors:** Frhana Rahmat, Jo Yee Kuan, Z Hajiman, Nik Noorul Shakira Mohamed Shakrin, Nur Aishah Che Roos, Marami Mustapa, Nur Adnin Ahmad Zaidi, Azimah Ahmad

**Affiliations:** 1 *Faculty of Medicine and Health Defence, Universiti Pertahanan Nasional Malaysia, 57000 Sungai Besi, Kuala Lumpur, Malaysia. *; 2 *Gnosis Laboratory (M) Sdn Bhd., No 1, Jalan USJ 21/11, USJ 21, 47630 Subang Jaya, Selangor, Malaysia. *

**Keywords:** Cervical cancer, human papillomavirus, prevalence- HPV 52, vaccination

## Abstract

**Background:**

Cervical cancer is the third leading cause of death in Malaysia, and Human Papilloma Virus (HPV) is the principal aetiology that is responsible for its development. This study was aimed to determine the prevalence and distribution of HPV types among different age groups, ethnicity, and areas in Malaysia.

**Materials and Methods:**

A total of 764 women aged 20-74 years old within the cities of Johor Bahru, Kuala Lumpur, Ipoh, Penang, and Kota Kinabalu underwent both cervical cytological assessment and HPV DNA analysis. Cervical cytology glass slides were prepared using the liquid base technique (Path TEZT TM). HPV DNA was extracted using TANBead^®^ Nucleic Acid Extraction Kit (Taiwan Advanced Nonotech Inc.), then the types were further identified using a DR.HPV Genotyping IVD kit.

**Results:**

The prevalence of HPV infection was 14.0% (107/764) with high-risk type at 10.7% (82/764) and low-risk type at 3.27% (25/764). The most common high-risk HPV types were HPV-52, 66, 33, 39, and 58 whereas low-risk HPV types were HPV-6, 40, and 81. The majority of HPV infections (80.37%) were detected in women with normal cytology results. The most prevalent HPV type among Chinese is 33 (n=6) followed by 16, 44, 58, 66 and 68 (n=5). Among Malays, HPV 16 and 51 were the two most prevalent types (n=2). The sensitivity of the HPV DNA test compared to cytology was 100% with a specificity of 88.37%.

**Conclusion:**

This study revealed that the most common high-risk HPV type among women living in urban areas in Malaysia is HPV 52, unfortunately which is not the type of infection the current HPV vaccine is covered for protection among females. These findings may contribute beneficial information to health care providers for the appropriate use of HPV vaccine in the prevention of cervical cancer in Malaysia.

## Introduction

Cervical cancer is the fourth leading cause of mortality among women worldwide, with an approximate 311,000 deaths and 570,000 new cases in 2018 (Arbyn et al., 2020). It is the most common cancer among women in under-developed countries due to inadequate effective control measures (Garland et al., 2008). Meanwhile, in developed countries, cervical cancer is uncommon due to effective education strategies, advanced screening programs, and availability of pre-cancerous treatment which reduce the incidence of mortality related to cervical cancer (Vu et al., 2018). In Malaysia, it is the third leading cause of female cancer deaths and the second most common cancer that occurs in women aged 15 to 44 years old with 621 death annually (Bruni et al., 2019). 

Human Papillomaviruses (HPV) are small non-enveloped viruses, with a double-stranded circular DNA genome. (Pinidis et al., 2016). There are more than 100 HPV types, and they are classified into high- and low-risk groups. The high-risk group is an oncogenic and persistent infection in the human system responsible for the development of cancer. Meanwhile, the low-risk group is involved in the development of genital warts which are benign growth on the cervix (Husain and Ramakrishnan, 2015). The two most important proteins in the carcinogenesis of cervical cancer are E6 and E7, which are responsible for the dysregulation of the cell cycle (Pinidis et al., 2016). HPV remains as one of the most prevalent causative agents for cervical cancer (Lowy and Schiller, 1998). Other risk factors attributed to the persistent HPV infection include unsafe sexual practices, smoking habits, and long-term use of oral contraceptives (Smith et al., 2003; Cancer, 2006; Žitkutė and Bumbuliene, 2016). 

The distribution of HPV types varies depending on geography and other environmental factors (Hamzi Abdul Raub et al., 2014). Worldwide, HPV16, HPV18, HPV33, HPV45, and HPV31 are the most prevalent types causing cervical cancer (Mutombo et al., 2019). In India, HPV 16 and 18 account for about 70% of all infections among Indian women (Diaz et al., 2008). The HPV 58 is rare worldwide but notably is significant in East Asia and part of Latin America. The possible reason is attributed to the variation in the distribution of HPV58 E6 and E7 (Chan et al., 2013). According to Bosch et al. (1995) and Barzon et al. (2008) different sexual habits and migration of people also may contribute to the heterogenous distribution of HPV types. In addition, environmental, cultural, or genetic factors also have impact in the HPV types distribution in various geographical area (Orozco-Colín et al., 2010). 

The HPV vaccine program was implemented in Malaysia in 2010 as a preventive measure against HPV infection among young generations. According to the Ministry of Health (MOH) 2011 guideline, the vaccine proposed for all MOH hospitals and clinics is a bivalent type (Cervarix®), which may protect against HPV type 16 and 18 infections. The other vaccine is Gardasil®, the tetravalent or quadrivalent type which has been used in private practices (general practitioners). This vaccine protects against HPV 16, 18, 6, and 11 infections. However, more studies are needed to show the true distribution of HPV group in different geographical part of Malaysia. This is crucial in assisting our Ministry of Health to implement a better type of HPV vaccine to be use in our population. Thus, this study was conducted to determine the prevalence and distribution of HPV types (low and high-risk) among women from urban areas in Malaysia. Numerous studies, meta-analyses and pooled analyses have established that HPV DNA test has better performance than cervical cytology screening in detecting high-grade cervical lesion (de Kok et al., 2012). Therefore, the present study also aimed to compare the performance of cervical smear and HPV DNA test in detecting HPV infections. Data obtained from this study is essential for updated information on the common HPV infection among women from urban areas in Malaysia. These findings may provide valuable information for the health care providers for the estimation of the recent HPV vaccine capability in the prevention of cervical cancer in Malaysia. 

## Materials and Methods

*Study design and samples*

The present study is a retrospective study and was conducted at Gnosis Laboratory Sdn. Bhd. This private laboratory was selected because it covers most of the urban areas in Malaysia, including Kuala Lumpur, Selangor, Johor Bahru, Ipoh, Penang, and Kota Kinabalu. All HPV DNA results were retrieved from archives in the Lab Information System (LIS) of Gnosis Laboratory from January 2017 to December 2018.

*Sampling procedures*

A total of 764 women who consented to participate in cervical cancer screening tests were included in this study between January 2017 and December 2018. The eligible criteria were all women who underwent both cervical cytological assessment and HPV DNA analysis from January 2017 to December 2018. Women who only requested either cytology screening or HPV DNA test only were not included in this study. The status of the HPV vaccine on these women is not known.

*Cervical cytology assessment*

This test was performed by first collecting cervical cell specimens from the endocervical canal of the subjects with a sterile Path TEZT TM Cervical Brush (Biocytech, Malaysia). Once the specimens arrived at the laboratory, the sample vial was placed into a PathTezt™ Processor and a gentle dispersion step breaks up blood, mucus, non-diagnostic debris, and thoroughly mixes the cell sample. The cells were then collected on a Filter specifically designed to collect diagnostic cells. The PathTezt™ Processor constantly monitors the rate of flow through the Filter during the collection process in order to prevent the cellular presentation from being too scant or too dense. A thin layer of cells was then transferred to a glass slide in a 20 mm-diameter circle, and the slide is automatically deposited into a fixative solution. Finally, the slides were assessed by cyto-screeners and pathologist before the diagnosis was acquired.

*DNA extraction*

Total DNA was isolated from the residual of cell suspension, by using TANBead® Nucleic Acid Extraction Kit (Taiwan Advanced Nonotech Inc.). DNA isolation was performed according to the manufacturer’s instructions. The concentration and purity of the DNA obtained were determined by using a spectrophotometer, and the quality of the DNA was examined with agarose gel electrophoresis. The total DNA isolated was stored at -20^o^C until further analysis.

*HPV genotyping*

HPV genotyping was carried out using a DR.HPV Genotyping IVD kit (DR. Chip Biotechnology Incorporation, Taiwan). The kit is an in vitro diagnostic assay for the detection and identification of at least 27 HPV types including 15 high-risk and 12 low-risk HPV types. The list of HPV according to the types:

• High-risk type: 16/18/26/31/33/35/39/45/51/52/53/55/54/56/58/59/66/68/69/70/72/73/82

• Low-risk type: 6/11/40/42/43/44/61/62/81/84

• Undetermined-risk type: 83

Firstly, the HPV gene fragment was amplified with a specific primer set provided in the kit. The reaction volumes and conditions were based on the manufacturer’s protocol. PCR was performed using a thermal cycler consisting of 35 cycles of amplification (95^o^C for 30 seconds, 50^o^C for 30 seconds, 72^o^C for 50 seconds). The initial denaturation cycle was carried out at 95^o^C for 10 minutes, and the elongation cycle was at 72^o^C for 7 minutes. Sample amplification control, positive control, and negative control were included in every batch of the sample run. 

After amplification, the HPV DNA amplicons were hybridised with an oligonucleotide array that was pre-spotted on DR. HPV Chip (DR. Chip Biotechnology Incorporation, Taiwan). Then, colorimetric signal development was performed using buffers and reagents provided in the kit. Lastly, the colorimetric development and signal were captured and analysed by DR. Aim Reader and DR. Aim Soft (DR. Chip Biotechnology Incorporation, Taiwan). The procedures were based on the manufacturer’s instructions. The assay was considered valid only if the sample amplification control, positive control, and hybridisation control signals were present, which respectively indicated a successful PCR reaction and probe hybridisation.

## Results

Table 1 shows the demographic data of women who consented to this study. The age of the participants ranged from 20 to 74 years, with a mean of 43.07 (SD±10.19). The participants were then sorted into four different age groups: < 21 years (0.4%), 21 to 29 years (11.5%), 30 to 65 years (86.6%) and above 65 years (1.4%). Most of the participants were Chinese (73.4%), followed by Malay (12.7%), other ethnicities such as Kadazan and Bajau (6.0%), and 5.1% participants were Non-Malaysians (Table 1). Almost half of the participants involved in this study were from Ipoh (45.4%), while the rest were residents of Kuala Lumpur (17.0%), Johor Bahru (12.2%), Kota Kinabalu (11.1%), and Penang (1.2%). 

A total of 764 cervical cell specimens were subjected to HPV DNA amplification. From the total samples screened, 107 samples (14%) were identified positive for both high-risk and low-risk HPV infections. Most of the HPV-positive participants were in the age group of 30-65 years old (86.6%). Most HPV infections were detected among Chinese participants (75.7%) followed by Malays (13.1%) and Indians (1.9%). Meanwhile, only 5% of HPV infection was present in both other ethnicities and non-Malaysians. Half of HPV prevalence was noted among participants from Ipoh (52.3%) followed by Kota Kinabalu (22.4%), Kuala Lumpur (14.0%), and Johor Bahru (11.2%). There was no HPV infection detected among Penang participants. 

**Figure 1:**
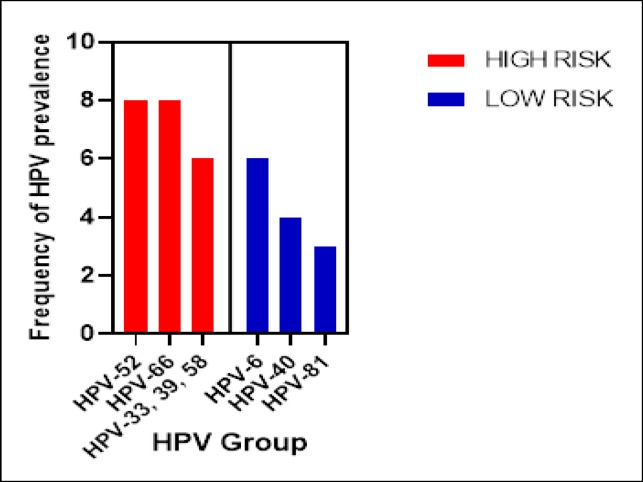
Commonest High-risk and Low-risk HPV Infection

Of 107 HPV-positive samples, 99 samples (89.4%) were associated with a single infection, and only 14 samples (10.6%) were related to multiple infections. The prevalence of high-risk HPVs was 10.7% (82/764) in which HPV-52 and 66 were the most common types (n=8) followed by HPV-33, 39, and 58 (n=6). A total of 3.27% (25/764) of low-risk type was detected, in which HPV-6 (n=6), HPV-40 (n=4), and HPV-81 (n=3) were the most common types (Figure 1).

**Figure 2:**
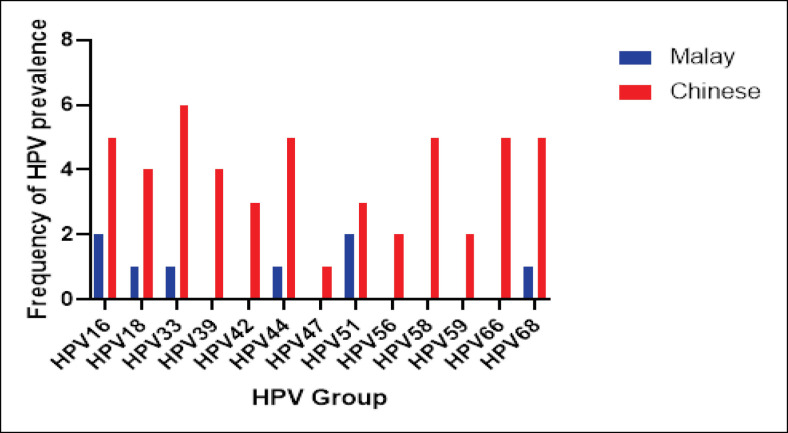
Distribution of High-risk HPV Types According to Ethnicity

Figure 2 presents the distribution of high-risk HPV types according to the two significant ethnicities involved in this study. It is shown that the most prevalent HPV type among Chinese is HPV 33 (n=6) followed by 16, 44, 58, 66, and 68 (n=5). Among Malays, HPV 16 and 51 are the two types with the highest prevalent (n=2).

The majority of HPV infections (80.37%) were detected among women with a negative cytological result for intraepithelial lesion or malignancy (NILM). Twelve cases (11.21%) were identified among an atypical squamous cell of undetermined significance (ASCUS), one case (0.93%) from an atypical squamous cell, cannot exclude HSIL (ASC-H), five cases (4.67%) from the low-grade squamous intraepithelial lesion (LSIL), two cases (1.87%) from the high-grade squamous intraepithelial lesion (HSIL) and one case (0.93%) from the unsatisfactory case (Table 2). Among the low-risk HPV infections, none of the cases revealed marked abnormal cytological results such as ASC-H, LSIL, and HSIL. In contrast, high-risk HPV infections were encountered in many abnormal cytology cases (Table 3). Five abnormal cytology cases were associated with multiple infections. Most abnormal cytology was from the ASCUS group and only one case from the ASC-H group. Five and two cases of high-risk infection showed LSIL and HSIL cytology results, respectively. 

The most common high-risk HPV types associated with abnormal cytology results were HPV 16,39, 51, and 68. The prevalence of HPV 18 was found only in two patients with abnormal cytology. Our data showed most of the high-risk HPV infections were from ASCUS group (n=9), followed by LSIL (n=5), HSIL (n=2) and ASC-H (n=1).

In the present study, the HPV DNA test was performed using the DR.HPV Genotyping IVD kit. The performance of this kit showed 100% sensitivity and 88.37% specificity with 18.87% PPV and 100% NPV (Table 4).

**Table 1:** The Demographic Data on Characteristics of the Study Subjects, Including their Age, Ethnicity, and Region

Variables	HPV test result n (%)		Total n (%)	p value*
	Positive	Negative		
Age group				
<21 years old	1	2	3 (0.4)	>0.05
21 – 29 years old	14	74	88 (11.5)	>0.05
30 – 65 years old	92	570	662 (86.6)	>0.05
>65 years old	0	11	11 (1.4)	>0.05
Ethnicity				
Malay	14	83	97 (12.7)	>0.05
Chinese	81	480	561 (73.4)	>0.05
Indian	2	19	21 (2.7)	>0.05
Others	5	41	46 (6.0)	>0.05
Non-Malaysian	5	34	39 (5.1)	>0.05
Urban Areas				
Kuala Lumpur	15	115	130 (17.0)	>0.05
Penang	0	9	9 (1.2)	>0.05
Ipoh	56	291	347 (45.4)	>0.05
Johor Bahru	12	81	93 (12.2)	>0.05
Kota Kinabalu	24	161	85 (11.1)	>0.05
Total	107	657		

HPV, Human papillomavirus; *p value <0.05

**Table 2:** HPV Positivity According to Cytological Diagnosis

HPV type	Cytological results						Total
	NILM	ASCUS	ASC-H	LSIL	HSIL	Unsatisfactory	
Low-risk	21	3	0	0	0	1	25
High-risk	65	9	1	5	2	0	82
Total	86	12	1	5	2	1	107

NILM, negative for intraepithelial lesions or malignancy; ASCUS, atypical squamous cells of undetermined significance; ASC-H, Atypical squamous cells, cannot exclude HSIL; LSIL, low grade squamous intraepithelial lesion; HSIL, high grade squamous intraepithelial lesion

**Table 3:** Distribution of High-risk HPV in Abnormal Cytological Diagnosis

High-risk HPV	Cytological diagnosis			
	ASCUS	ASC-H	LSIL	HSIL
	(n)	(n)	(n)	(n)
16	-	-	(1)	-
16,18,68	(1)	-	-	-
16,44	(1)	-	-	-
18	-	-	-	(1)
33		-	-	(1)
39	(2)	-	-	-
39,47	(1)	-	-	-
42,59	(1)	-	-	-
44,51	-	-	(1)	-
51	(1)	-	(1)	-
56	(1)	-	-	-
58	-	-	(1)	-
66	(1)	-	-	-
68	-	(1)	(1)	-

HPV, Human papillomavirus; ASCUS, Atypical squamous cell of undetermined significance; ASC-H, an atypical squamous cells, cannot exclude HSIL; LSIL, low-grade squamous intraepithelial lesion; HSIL, high-grade squamous intraepithelial lesion.

**Table 4:** Performance of DR. HPV Genotyping IVD in Detecting HPV DNA

		Cytological test		Sensitivity (%)	Specificity (%)	PPV	NPV
		Positive	Negative				
HPV DNA	Positive	20	86	100%	88.37%	18.87%	100%
	Negative	0	654				

HPV DNA, Human papillomavirus deoxyribonucleic acid; Sens, sensitivity; Spec, specificity; PPV, positive predictive value; NPV, negative predictive value.

## Discussion

Globally, HPV 16 and 18 are the most common types that are responsible for the development of cervical cancer and pre-cancerous cervical lesions (Bray et al., 2018). In Asian geographic regions, the HPV 16/18-positive fraction accounted for almost 70% of women with cervical cancer (Bao et al., 2008). However, the distribution of HPV types is heterogeneous, depending on the geography and other environmental factors (Hamzi Abdul Raub et al., 2014). 

The present study is the first in Malaysia that used large-scale data collected from a private laboratory to determine HPV prevalence among women living in urban areas in Malaysia. This study covered mainly urban areas in Peninsular Malays mainly of the age group of 21-65 years old, possibly due to the recommendation from the Malaysian National Guidelines on Pap Smear Screening that all sexually active women aged 20–65 years should attend screening annually for two consecutive years and subsequently every three years if the results are normal. Although Malay is the largest ethnic group in Malaysia; however, in our study, most of the participants were Chinese. According to Abdullah et al., (2013), non-Malays utilized more of the private facilities that require higher cost than governmental health centers because patients attending government clinic encountered problems with long waiting time, overcrowding and limited parking space.

The prevalence of HPV infection reported in this study was 14%, slightly higher than what was found in other studies in Malaysia which was 9.6% and 7.2% (Khoo et al., 2018; Sainei et al., 2018). Khoo et al., (2018) carried out a large-scale study involving five community-based clinics in Selangor, which revealed that high-risk HPV prevalence was 6.5%. Our data showed that the prevalence of high-risk HPV was higher (10.7%). The observation might be due to the different ethnicity cohorts among the two groups, whereby in the study by Khoo et al., (2018) majority of participants were Malays. In addition, a previous study reported that the Chinese have the highest prevalence of HPV infection compared to other races in Malaysia (Hamzi Abdul Raub et al., 2014). However, the data obtained from our study may not reflect the entire Malaysian population since only urban areas were covered. 

Two main ethnicities involved in our study were Malays and Chinese with different types of HPV infection. Hamzi Abdul Raub et al., (2014) reported that the percentage of HPV 16 infection was significantly higher in Chinese (75.9%) compared to Malays (63.7%) while HPV 18 was significantly higher in Malays (52.6%) compared to Chinese (25.0%). Concurrently, HPV 33 and 52 were also more commonly detected in the Chinese. The finding is supported by Magnusson et al., (2000) which suggested that predisposing genetic factors play a vital role in various steps of carcinogenesis, namely sensitivity to or persistence of HPV infection, as well as the rate of tumour development. 

There are more than 100 types of HPV which are further divided into high-risk (HR) and low-risk (LR) groups. HPV types 16 and 18 are considered as the most prevalent HR types, whereas the 6 and 11 are LR types (Husain and Ramakrishnan, 2015). In our study, we found that the most prevalent HR group are HPV 52 and 66, followed by HPV 33, 39, and 58. Our finding is similar to Peng et al., (2012) who found HPV 52 was the most prevalent type in South-East Asia (12.9%), followed by HPV 16 (8.5%), 58 (5.2%), 18 (5.0%), and 66 (4.9%). In addition to HPV types 16 and 18, type 52 and 58 were also frequently observed in invasive cervical carcinoma (ICC) among Asian women (Quek et al., 2012). A study by Ezat et al., (2010) revealed that the three most typical HR-HPV types in Cervical Intraepithelial Neoplasia (CIN) smears are HPV 16, 51, and 52. While another study reported that the two most common HPV types among cervical cancer patients in the North-Eastern region of West Malaysia are HPV 16 and 58 (Othman and Othman, 2014). For the LR group, the most prevalent type found in our study were HPV 6, followed by 40 and 81.

Based on a previous meta-analysis report, the global prevalence of cervical HPV infection among women with normal cytological smear result was 12% (Chan et al., 2019). Another meta-analysis revealed that the prevalence of cervical HPV infection in normal cytology among women from Asia was 14.4% (Bao et al., 2008). Conversely, our data showed the prevalence of HPV infection among normal cytology smear result was 80.37%. The vast differences in the estimates can be due to several factors such as geography, and the method used to detect HPV. An extensive meta-analysis study done by Bruni et al., (2010) involving one million subjects from five continents found that, the prevalence of HPV infection among women with normal cytological findings was heterogenous by geographical region. They reported that European, Northern American, and Asian regions showed lower HPV prevalence than African and South American continents. Two studies were carried out in 2001 and 2008, using PCR with different detection methods on the same participants in the rural area of Mozambique found a diverse HPV prevalence of 32.1% and 76.0%, respectively (Castellsagué et al., 2001; Castellsagué et al., 2008). Thus, showed that the sensitivity and specificity of PCR-based method can influenced the estimation of HPV infection. In addition, Bruni et al., (2010) found that there were a large variability in HPV prevalence ranging from 2.9% to 80.8% among 19 studies that were carried out in the same country and region within the United States. 

In this study, HPV 51 was found to be among the most prevalent HPV types that were associated with abnormal cytological finding. This can be due to that the highest participants in this study were Chinese. Hamzi Abdul Raub et al., (2014) reported that HPV 51 are prevalence among Chinese in Malaysia. Simultaneously, Piana et al., (2013) revealed that HPV 51 has a very clear correlation with cervical cancer. In addition, a few studies have reported that prevalence of HPV 51 is higher in the patient with HIV infection (Sahasrabuddhe et al., 2007; Mane et al., 2012; Zhang et al., 2012). 

Human-papillomavirus (HPV) DNA testing has been nominated as an alternative to primary cervical cancer screening using cytological testing. In the present study, the performance of HPV DNA test using the DR.HPV Genotyping IVD kit had 100% sensitivity with 88.37% specificity. In addition, molecular methods for HPV detection have excellent performance which provide almost 100% negative predictive value in detecting the prevalence of high-grade lesion (Coutlée et al., 2005). Our finding is concurrent with other numerous studies which reported that HPV DNA test is superior to cytological or Pap test as cervical screening tool (de Kok et al., 2012; Hamzi Abdul Raub et al., 2014; Pileggi et al., 2014).

There are some limitations to our study. Firstly, it does not reflect the actual ethnicity breakdown of the country. Having Chinese individuals, as the high number of women who participated in cervical cancer screening, prevented us from concluding the actual prevalence of cervical cancer in Malaysia. Secondly, data was mainly obtained from private clinics whereby mainly Chinese women who can afford for the high cost compared to Malay women. Thirdly. we do not have data on cervical biopsy, which is the gold-standard test for the diagnosis of pre-malignant as well as cervical cancer.

In conclusion, this study revealed that the most typical high-risk HPV type among women from the urban areas in Malaysia is HPV 52 that is unfortunately not covered by the current HPV vaccine administered in Malaysia. These findings may contribute beneficial information to health care providers for the estimation of the recent HPV vaccine capability in the prevention of cervical cancer in Malaysia. The future generation HPV prophylactic vaccine should also include HPV 52, 66, 58, 33, and 39 types to achieve efficient vaccine coverage.

## Author Contribution Statement

All authors read, critically reviewed and endorsed the final manuscript. JKY and HZ: Data extraction. SNNSM: Advise on laboratory tests and critical revision of manuscript. CRN, AA and ANA: Data collection, statistical analysis and its interpretation. MM and RF: Literature review, discussion and manuscript writing. 
